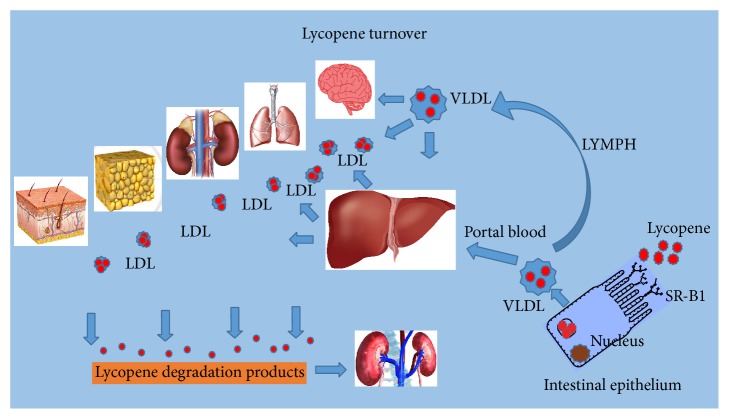# Lycopene Deficiency in Ageing and Cardiovascular Disease

**DOI:** 10.1155/2016/3218605

**Published:** 2016-01-06

**Authors:** Ivan M. Petyaev

**Affiliations:** Lycotec Ltd., Granta Park Campus, Cambridge CB21 6GP, UK

## Abstract

Lycopene is a hydrocarbon phytochemical belonging to the tetraterpene carotenoid family and is found in red fruit and vegetables. Eleven conjugated double bonds predetermine the antioxidant properties of lycopene and its ability to scavenge lipid peroxyl radicals, reactive oxygen species, and nitric oxide. Lycopene has a low bioavailability rate and appears in the blood circulation incorporated into chylomicrons and other apo-B containing lipoproteins. The recent body of evidence suggests that plasma concentration of lycopene is not only a function of intestinal absorption rate but also lycopene breakdown via enzymatic and oxidative pathways in blood and tissues. Oxidative stress and the accumulation of reactive oxygen species and nitric oxide may represent a major cause of lycopene depletion in ageing, cardiovascular disease, and type 2 diabetes mellitus. It has been shown recently that low carotenoid levels, and especially decreased serum lycopene levels, are strongly predictive of all-cause mortality and poor outcomes of cardiovascular disease. However, there is a poor statistical association between dietary and serum lycopene levels which occurs due to limited bioavailability of lycopene from dietary sources. Hence, it is very unlikely that nutritional intervention alone could be instrumental in the correction of lycopene and carotenoid deficiency. Therefore, new nutraceutical formulations of carotenoids with enhanced bioavailability are urgently needed.

## 1. Introduction

Lycopene is a polyunsaturated hydrocarbon phytochemical present in red fruit and vegetables (papayas, tomatoes, red peppers, watermelons, etc.) and belongs to the tetraterpene carotenoid family [1]. Ingestion of products containing cooked tomato accounts for about 80% of daily dietary intake of lycopene in the developed world [2, 3]. Despite significant individual disparities, the average consumption of lycopene varies between 5 and 7 mg/day in the western world [4]. Due to its distinctive ability to neutralize free radicals, lycopene is believed to confer measurable protection against cancer, atherosclerosis, diabetes, and some inflammatory diseases [5, 6]. Indeed, a growing body of epidemiological evidence suggests that lycopene consumption is associated with decreased risk of various chronic diseases, while lycopene also demonstrates significant antioxidant activity in a number of in vitro and in vivo systems [7]. The multiple biological effects of lycopene are predetermined by the unique chemical structure of the compound and its particular physicochemical properties.

## 2. Physical and Chemical Properties

Lycopene is a 40-carbon atom acyclic fat-soluble compound containing 13 linearly aligned double bonds, 11 of them being conjugated. It occurs in nature as an all*-trans*-isoform, often referred to as* all-E*-lycopene [8]. Thermal processing as well as intestinal digestion of raw tomato products facilitates* cis-*isomerization of lycopene [9]. In the human body, lycopene is represented predominantly by various* cis*-isomers (referred to as (*Z*)-lycopene) suggesting that* cis*-transformation is essential for efficient intestinal absorption [10].* cis*-isomerization can also be initiated by exposure of lycopene to heat, light, and oxygen although this can eventually cause irreversible degradation of the lycopene molecule to a number of small end products [8]. C=O bonds are chromogenic and confer a distinctive red color to lycopene crystals. Double bonds are essential to the antioxidant properties of lycopene which are a major functional feature of the compound. Amongst the different carotenoids, lycopene has the highest ^1^O_2_ quenching ability, exceeding the antioxidant properties of carotene by at least twofold [11]. (*Z*)-isomers have the greatest antioxidant activity in scavenging lipid peroxyl radicals [8, 11].

## 3. Bioavailability and Absorption

Bioavailability of lycopene and other carotenoids is poorly understood and studied. It has been shown recently that structural localization of lycopene in the chloroplasts of fruit and vegetables is an important factor limiting bioavailability of lycopene from dietary sources since chloroplasts have high resistance to gastric and intestinal digestion [10]. Thus, food matrix structure predetermines significantly the bioavailability of lycopene. A significant portion of dietary lycopene is excreted from the human body in undigested form and there is no immediate absorption peak in plasma lycopene level after a single tomato meal. However, there is a clear cumulative lycopene absorption spike in the plasma of volunteers after a 5-day ingestion period of lycopene-containing products [12]. As shown in Figure 1, the ingested portion of lycopene released from the food matrix becomes solubilized and emulsified inside the intestinal lumen and is transported with scavenger receptor class B type 1 protein (SR-B1) via the epithelium of small intestine [13]. Lycopene distribution among tissues is very selective. The testes, adrenals, liver, and prostate have the highest lycopene concentration, while other organs are known to have much lower lycopene content [14]. Plasma lipoproteins are major delivery vehicles of carotenoids and lycopene in the human body [15].

Therefore, it is very likely that the differences in lycopene tissue levels are related to variation in tissue expression of lipoprotein receptors and cholesterol transporters.

## 4. Daily Requirements 

There is no consensus on recommended daily dose of lycopene since carotenoids are considered to be nonessential micronutrients. In the industrialized European countries, daily intake of lycopene varies from 0.7 mg (Finland) to 1.3 mg (Germany), while a much higher range (3.7–16.1 mg) is reported for the United States [16]. Higher intake of lycopene at daily dosage up to 100 mg has no side effects in volunteers [17]. No evidence of toxicity of lycopene has been obtained from in vivo studies using laboratory animals [18]. Clinical studies use moderate amounts of lycopene rarely exceeding 10 mg per day. However, in animal experiments daily supplementation up to 200 mg/kg has been reported [19]. Therefore, low toxicity and high tolerance of lycopene open the door to various options in the design of lycopene supplementation protocols.

## 5. Lycopene in Blood

Factors influencing blood levels of lycopene represented in humans by* cis*-isoforms are not well understood as yet. Among these is geographic location of individuals and health status as well as a number of sociodemographic factors. It has been reported [20] that median lycopene concentration in the plasma of the general population of the USA is 0.59 *μ*mol/L (range 0.07–1.79), whereas average lycopene concentration in the Scandinavian countries, in particular Finland, seems to be lower −0.16 ± 0.11 *μ*mol/L. A very recent study [21] defines world regions with low and high levels of lycopene consumption. Surprisingly, Northern and Western Europe along with Central Africa and the Middle East represent the geographical areas with the lowest lycopene consumption as opposed to the Asian countries which display the highest level of lycopene consumption in the world attributed to high intake of fruit and vegetables [21]. It is also known that age and plasma lipid levels (LDL, total cholesterol, and triglycerides) are inversely correlated to blood lycopene level [20, 22]. Lower lycopene values are reportedly associated with being unmarried, of lower income, and an older nonwhite male [20].

## 6. Lycopene Depletion

Recent developments in molecular medicine reveal that the plasma level of lycopene and other carotenoids is a function of intestinal absorption rate as well as lycopene utilization in the reactions of biological oxidation in tissues. The antioxidant properties of lycopene and its ability to scavenge lipid peroxyl radicals are attributed to the eleven conjugated double bonds between its carbon atoms. When double bonds are oxidized and broken by reactive oxygen and reactive oxygen species, the lycopene molecule undergoes irreversible nonenzymatic degradation leading to the formation of various oxidative metabolites such as 2-apo-5,8-lycopenal-furanoxide, lycopene-5,6,5′,6′-diepoxide, lycopene-5,8-furanoxide, and lycopene-5,8-epoxide isomers [23, 24]. On the other hand, lycopene level in blood and tissues can be significantly affected by enzymatic degradation leading to the formation of different end products. In particular, enzymatic cleavage of lycopene by lipoxygenase is accompanied by accumulation of 3-keto-apo-13-lycopenone and 15,15′-apo-lycopenal among other minor cleavage products [24]. Depletion of lycopene as well as formation of lycopene oxidative metabolites can be reproduced in cell-free plasma specimens and isolated lipoproteins by introducing into the medium enzymatic systems generating reactive nitrogen species [25].

Taken together, these results suggest that oxidative stress as well as hyperactivity of the endogenous enzymes responsible for generation of reactive oxygen species and nitric oxide may deplete lycopene reserves in human cells and tissues.

## 7. Lycopene Deficiency in Ageing

Plasma lycopene level can become significantly reduced during the process of ageing. Older individuals show statistically lower lycopene concentration values in blood as compared to younger matching individuals with similar ethnic and dietary background [26]. Intestinal absorption of carotenoids is a complex multistage process which requires a fully intact and functioning gastrointestinal epithelium and subset of various enzymes [27]. Acute and chronic gastritis and abnormal gastric acid secretion as well as deviations in intestinal enzyme spectrum during ageing are considered to be major causes of reduced intestinal carotenoid absorption in older individuals [28]. Moreover, depleted levels of lycopene and other carotenoids in older individuals are believed to reflect age-related changes in the intestinal microbiota, which regulates bioavailability of carotenoids and polyphenols in the large intestine [29]. Although additional research to explain the causes and mechanisms of lycopene deficiency in ageing is required, it is clear now that correction of carotenoid deficiency in older individuals may have an enormous impact on their health status. Recent DNA microarray analysis reveals that lycopene supplementation prevents transcriptional activation of genes implemented in the process of ageing in multiple strains of mice [30]. This antagonizing action was as effective as the effect of well-known SIRT-1 activators such as starvation and resveratrol. Moreover, lycopene has recently been shown to suppress activation of the mTOR/AMPK cascade, a major metabolic pathway linked to ageing [31]. These cutting-edge observations help us to understand the multiple clinical and experimental reports revealing the effects of lycopene on a variety of health conditions associated with ageing. There are numerous pieces of clinical evidence suggesting that lycopene supplementation prevents osteoporosis and incidence of bone fractures [32, 33], improves pulmonary function [34], delays skin ageing [35], and enhances physical performance in elderly patients [36]. The set of clinical consequences of lycopene deficiency is greatly enlightened by the solid body of clinical evidence revealing the crucial role of lycopene in maintaining prostate health and its ability to prevent prostate cancer in elderly males [37, 38].

## 8. Lycopene and Cardiovascular Disease

Cardiovascular disease (CVD) remains a leading cause of mortality and disability around the world. There is a massive body of epidemiological results suggesting that Mediterranean countries have a lower rate of CVD mortality when compared to other regions of Western Europe and the United States [39]. The reduced rate of CVD mortality can be explained, at least in part, by the dietary culture of the Mediterranean region which includes consumption of large amounts of fruit and vegetables. Tomato-based products represent an essential element of the Mediterranean diet, which motivates many researchers to search for the link between lycopene consumption and occurrence of CVD. Epidemiological studies provide indisputable evidence supporting the direct role of lycopene in prevention of CVD. As has been recently confirmed in the Framingham Heart Offspring Study [40], there is a strong inverse association between lycopene intake and incidence of myocardial infarction, angina pectoris, and coronary insufficiency. Low plasma lycopene levels were reported by many researchers in hypertension, myocardial infarction, stroke, and atherosclerosis [41, 42]. Less convincing results and a more complex landscape emerge when the data from interventional studies on lycopene use in CVD patients are analyzed. There are multiple conflicting reports on how lycopene administration affects the progression of CVD and its outcomes [43, 44]. However, there is a certain degree of reproducibility in scientific reports describing the reduction of cholesterol (LDL and total), upregulation of HDL [45], decrease in carotid artery intima-media thickness [46], and lowering of both plasma markers of oxidative damage [44] and postprandial oxidative stress [47] in patients treated with lycopene. Nevertheless, the effect of lycopene on progression of CVD remains a controversial topic in modern medical science and requires further well-designed clinical studies. The experimental approach brings much more certainty regarding the beneficial role of lycopene in CVD. There are multiple and reproducible reports describing normalization of endothelial nitric oxide synthase activity and nitric oxide level in coronary arteries [48], inhibition of the mevalonate pathway of cholesterol biosynthesis [49], improvement of endothelial function, and attenuation of inflammatory damage [50] as well as improvements in lipoprotein profile and their turnover [48] in different animal models of CVD. These changes may represent a molecular basis for lycopene action in CVD. Significant discrepancies in the outcomes of clinical and experimental studies verifying the effect of lycopene on CVD can be explained, in our opinion, by significant fluctuations in preexisting levels of lycopene in the blood of subjects enrolled on clinical trials, whereas lycopene concentration in the blood of experimental animals kept on standardized diets tends to stay in a similar range. However, individual variations in plasma lycopene level in general populations may have a significant impact on public health. As recently shown [51], low serum lycopene and total carotenoid levels predict all-cause mortality as well as poor outcomes and rapid progression of CVD in the adult population of the USA. The majority of individuals, even in developed countries, have astonishingly decreased levels of lycopene and total carotenoids in the blood which translates into higher stroke risk [52]. Decreased alpha-carotene and lycopene concentrations in the blood have recently been proposed as possible criteria for prognosis of public health since they are inversely associated with CVD mortality in a highly significant manner [53]. Although carotenoids are considered nonessential micronutrients, there have been some recent attempts to declare desirable plasma concentration levels of carotenoids for public health. As recently proposed, there are 5 cut-off levels for plasma carotenoid levels [54]. According to the results of meta-analysis, a plasma carotenoid level <1 *μ*M translates into a very high risk of health consequences. Moderate health risk is proclaimed to be associated with carotenoid concentration in the range 1.5–2.5 *μ*M. Values for carotenoid concentration from 2.5–4 *μ*M suggest a moderate risk, whereas carotenoid concentrations over 4 *μ*M are proposed to have the lowest risk of health consequences. According to the same report, over 95% of the US population falls into the moderate or high risk category of the carotenoid health index.

## 9. Conclusion

Recent advances in medical and analytical chemistry have allowed pinpointing of multiple nonalimentary diseases and pathological conditions associated with micronutrient deficiencies. It has been increasingly recognized that health conditions associated with oxidative stress (ageing, CVD, and type 2 diabetes mellitus) are accompanied by significant deviations in plasma and tissue levels of many important nutrients, in particular lycopene and other carotenoids [55, 56].

Lycopene is the most powerful antioxidant from the tetraterpene carotenoid family, its anti-radical properties being mediated by eleven conjugated double bonds between carbon atoms. Lycopene is essential in scavenging lipid peroxyl radicals, reactive oxygen species, and nitric oxide. Recent advances in molecular science reveal that the interaction of lycopene with reactive oxygen species leads to irreversible nonenzymatic degradation of lycopene and formation of various oxidative lycopene metabolites. There is also a newly identified enzymatic pathway of lycopene degradation that results in the formation of several end products excreted from the human body. Thus, health conditions accompanied by long-lasting oxidative stress may cause lycopene depletion and require constant and efficient replenishment of carotenoids in the antioxidant “basket” of human cells and tissues. However, lycopene from dietary sources has extremely low absorption and bioavailability rate. It appears in the blood circulation incorporated into chylomicrons and other apo-B containing lipoproteins and requires a sophisticated system of intestinal absorption and distribution among the tissues. Therefore, plasma concentration of lycopene needs to be considered as an integral value reflecting both the intestinal absorption rate of carotenoids and the intensity of lycopene breakdown via enzymatic and oxidative pathways in blood and tissues.

There is alarming prevalence of lycopene and carotenoid deficiency in older individuals and CVD patients as well as widespread carotenoid deficiency in the general population, as revealed by latest research. This illustrates the necessity for a well-designed nutritional strategy and new nutraceutical products capable of normalizing plasma lycopene and carotenoid levels in an efficient manner. However, it is very unlikely that nutritional intervention alone will be sufficient in the correction of carotenoid deficiency. There is a poor statistical association between dietary and serum lycopene levels which can be explained by the limited bioavailability of lycopene from dietary sources. As shown recently, plasma concentration of lycopene, but not dietary intake of lycopene, correlates with predisposition to stroke [53]. It is suggested that even a high intake of lycopene-containing food products may not affect the health outcomes of lycopene and carotenoid deficiency in the general population. Therefore, new nutraceutical formulations of carotenoids and lycopene with enhanced bioavailability are urgently needed. In recent years, new formulations of lycopene and other carotenoids with increased bioavailability have been created using microemulsifying protocols and/or nanodelivery systems [57, 58]. Their implementation could be a milestone development in the prevention and treatment of the health consequences of lycopene and carotenoid deficiency in the general population.

## Figures and Tables

**Figure 1:**